# Fibrous Dysplasia Meets Intramuscular Myxoma: Mazabraud Syndrome—First Documented Case in Pakistan

**DOI:** 10.1155/carm/3804294

**Published:** 2026-02-26

**Authors:** Alizah Faisal, Asfand Yar Ali, Hooria Waqas, Hania Masood, Muhammad Sheraz Hameed, Noor Ul Huda Al Hadi, Rahmat Gul Omarzai

**Affiliations:** ^1^ Department of Medicine, Rawalpindi Medical University, Rawalpindi, Pakistan; ^2^ Department of Pathology, Army Medical College, Rawalpindi, Pakistan, numspak.edu.pk; ^3^ Department of Medicine, Nangarhar Medical University, Jalalabad, Afghanistan

**Keywords:** bone deformities, fibrous dysplasia, intramuscular myxomas, limb shortening, Mazabraud syndrome

## Abstract

**Background:**

Mazabraud syndrome (MS) is a rare benign genetic disorder characterized by the coexistence of fibrous dysplasia (FD) and intramuscular myxoma (IM), with a very limited number of cases reported worldwide, and none has been documented in Pakistan until now. It typically presents in middle‐aged females and affects the lower limbs. Its pathogenesis is associated with activating mutations in the GNAS gene. This disease is often under‐recognized in low‐income countries. It can rapidly progress to bone deformities, fractures, and unnecessary aggressive treatments if not promptly recognized.

**Case Presentation:**

A 64‐year‐old male presented with progressive fatigue and pallor for three months, alongside a lifelong history of multiple bone deformities and painless soft tissue swellings. He had recurrent fractures since childhood, leading to severe limb deformities and dependence on crutches. Physical exam revealed bosselated swellings over the axilla, thigh, and groin, with significant limb shortening. Imaging showed widespread lytic lesions and necrotic soft tissue masses. Blood work revealed hypochromic microcytic anemia with rouleaux formation; white cell and platelet counts were normal. Bone marrow biopsy demonstrated Grade III myelofibrosis with characteristic “Chinese letter” trabeculae, confirming FD. Biopsy of soft tissue masses showed benign IMs. A diagnosis of MS was made based on the coexistence of polyostotic FD and multiple myxomas.

**Conclusion:**

MS is a rare and benign genetic disorder that requires a high index of suspicion for timely diagnosis. This unique case underscores the need for increased clinical awareness of MS in resource‐limited settings where genetic testing is not common and the importance of multidisciplinary evaluation for accurate diagnosis and management. Timely diagnosis and treatment can prevent bone deformities, fractures, and disabilities and improve patient outcomes for this uncommon syndrome.

## 1. Introduction

Mazabraud syndrome (MS) is characterized by fibrous dysplasia (FD) in either a single bone or multiple bones and is a rare benign genetic disorder associated with soft tissue myxomas [1]. The first ever case of MS was described by Henschen et al. in 1926, with a prevalence of < 1/1,000,000, and the link with FD was established by Mazabraud et al. in 1967 [2, 3]. Only 107 cases have been reported in the global literature (as of 2019) [4].

FD is a rare, benign skeletal disorder defined by the replacement of normal bone and bone marrow with abnormal fibro‐osseous tissue, resulting in fragile bones with structural deformities, pain, fragility fractures, and impaired mobility [4]. It accounts for 5%–7% of all benign bone tumors, with an incidence of approximately 1 per 30,000 individuals. FD commonly affects the long bones of the body, as well as the facial bones.

Intramuscular myxomas (IMs) are benign soft tissue neoplasms characterized by undifferentiated spindle‐shaped or stellate cells set within an abundant collagenous and myxoid extracellular matrix, having a reported incidence of 0.1–0.13 per 100,000 [5]. These lesions most commonly occur in large skeletal muscle groups, especially in the lower limbs (thighs and buttocks), and are often seen alongside bony deformities resulting from FD. They tend to be unilateral and can appear either concurrently or develop years apart. They are slow‐growing, typically painless tumors that primarily affect adults [6]. These tumors have a very low recurrence rate after surgical excision.

MS shows a 2:1 female‐to‐male prevalence ratio, indicating a higher incidence in females [7]. Although the exact pathogenesis remains unclear, activating mutations of the GNAS gene are the most common cause of this disease. The average age of diagnosis is 32 years, with FD usually diagnosed 12 years before myxomas [8]. Due to the scarcity of documented cases and limited research on the genetic basis and diagnosis of MS in Pakistan, the condition remains highly under‐recognized, highlighting the need for further investigation and increased clinical awareness. We report the first documented case of MS in Pakistan, presenting in a male patient with progressive pallor over the past 3 months and a long‐standing history of multiple bony deformities and soft tissue swellings since childhood.

## 2. Case Presentation

### 2.1. History of Presentation

**FIGURE 1:**
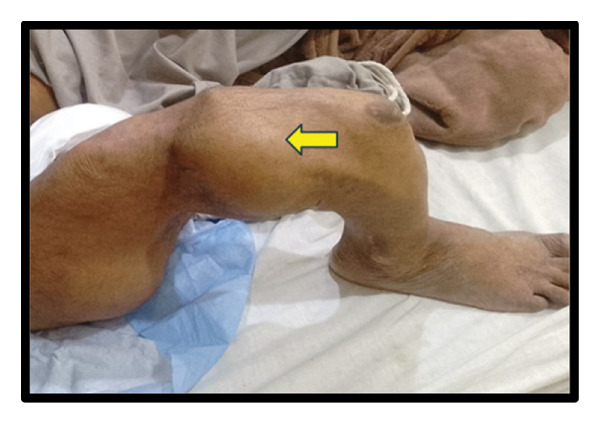
Clinical photograph showing a femoral fracture resulting in deformity of the right lower limb.

A 64‐year‐old man presented to Holy Family Hospital, Rawalpindi, on May 26, 2025, with complaints of increasing fatigue and pallor over the past three months. Alongside these symptoms, he had long‐standing bony deformities and soft tissue swellings, which he reported had been present since early childhood. His medical history revealed that around the age of seven, he sustained a femoral fracture following a trivial fall (Figure 1). Over the following years, he experienced repeated fractures involving the right femur, tibia, and humerus. These events led to progressive limb deformities, eventually resulting in significant disability. For the past several decades, he has relied on crutches for mobility and daily functioning.

In addition to his skeletal complaints, he noticed multiple painless swellings developing over the axilla, thigh, and groin regions, which he never sought treatment for until October 2024. At that time, he was presented to a local hospital in Sargodha due to an infected swelling in his right thigh. The patient also shared a notable family history: similar soft tissue swellings had occurred in his brother, son, and nephew, suggesting a possible hereditary component.

### 2.2. Physical Examination

On physical examination, the patient appeared pale and in mild distress. Vital signs were stable (BP: 120/90 mm/Hg; pulse: 78/min; and R.R: 12/min). The patient had visible limb deformities. Multiple bosselated swellings can be seen over the right arm and axilla (Figure 2(a)). There is also marked deformity of the right femur with a large soft tissue mass over the thigh (Figure 2(b)). Local examination of the thigh soft swelling revealed tenderness, warmth, and erythema. A prominent inguinal mass on the right side can also be appreciated (Figure 2(c)). Limb shortening and bowing were apparent, particularly in the right lower limb. No neurological deficits were present.

FIGURE 2(a) Multiple bosselated swellings over the right arm and axilla. (b) Marked deformity of the right femur associated with a large soft‐tissue mass over the thigh. (c) Prominent right‐sided inguinal mass on clinical examination.

**Figure:**
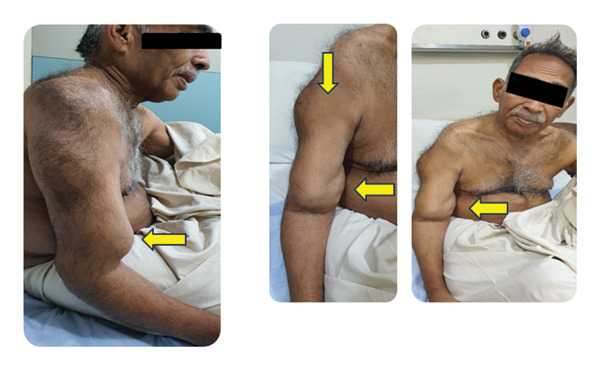
(a)

**Figure:**
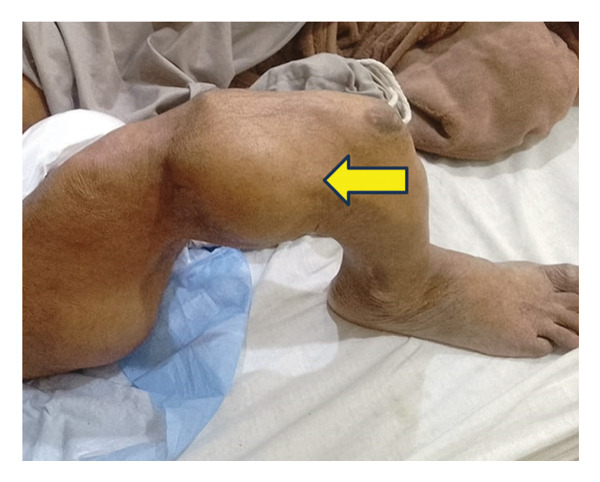
(b)

**Figure:**
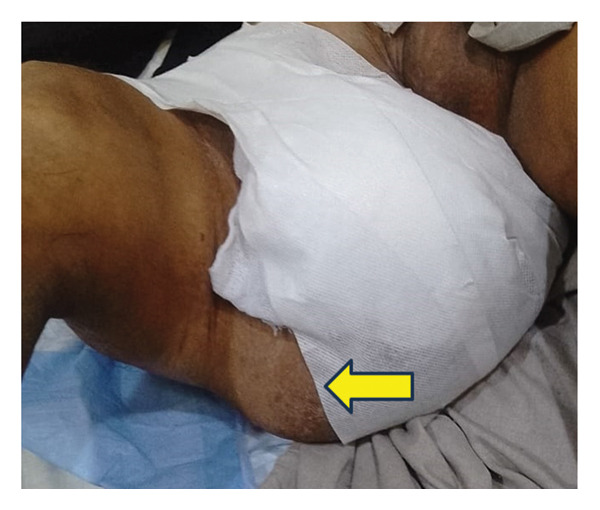
(c)

### 2.3. Radiological Examinations

**FIGURE 3:**
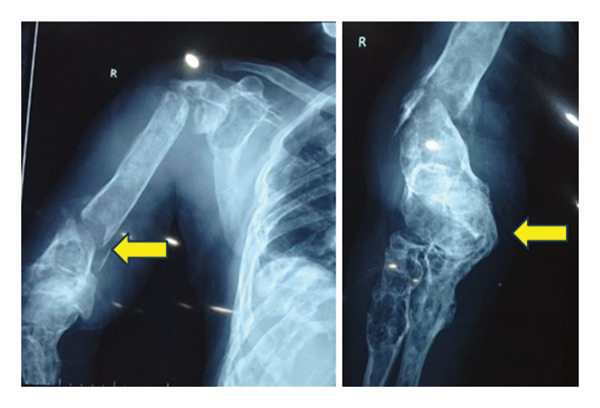
X‐ray of the right humerus demonstrating fractures at the proximal and distal diaphysis with abnormal bone formation.

X‐ray of the right arm showed fractures in both proximal and distal diaphysis of the humerus, radius, and ulna (Figure 3). CT scan revealed necrotic, peripherally enhancing soft tissue masses in the right axilla, displacing adjacent vessels. Multiple fluid collections with air loculi were present in both thighs, with surrounding inflammation and displacement of neurovascular structures. Diffuse punched‐out lesions were noted throughout the skeleton.

MRI demonstrated widespread expansile lytic lesions with cortical disruption, diffuse soft tissue cellulitis in the right gluteal and thigh regions, and a heterogeneously enhancing ulcerative lesion in the adductor compartment.

The Tc‐99m bone scan showed multifocal active bone lesions involving both axial and appendicular skeleton, along with extraskeletal uptake in the right thigh and left calf.

### 2.4. Laboratory Findings

**FIGURE 4:**
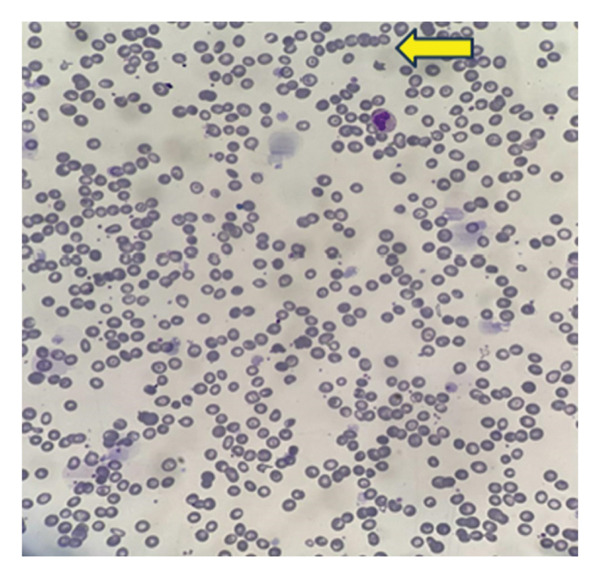
Peripheral blood smear showing microcytic hypochromic anemia with rouleaux formation (arrow indicates rouleaux).

TLC revealed normal WBCs with normal differential counts. CBC shows microcytic, hypochromic anemia, low hemoglobin, RBC count, MCV, and MCH, consistent with anemia of chronic disease. Elevated ESR (> 140 mm/hr) suggests significant underlying chronic disease (Table 1). RBC morphology revealed hypochromic microcytic anemia with rouleaux formation, and there were adequate platelets on the blood smear (Figure 4). Infection within the right thigh lesion was supported by a raised CRP. All other blood chemistry, viral markers, and autoimmune screening were within normal limits.

**TABLE 1:** CBC and ESR.

Parameters	Result	Unit	Reference range
RBC	2.86	× 10^12^/L	4.5–5.5
Hemoglobin	7.0	g/dL	13–17
MCV	77.6	fL	83–101
MCH	24.5	pg	27–32
TLC	6.48	× 10^9^/L	4–10
Platelet count	215	× 10^9^/L	150–410
ESR	> 140	mm at 1st hour	0–9

Based on the combination of these findings, there was suspicion of FD, and he was referred to the hematology department at AFIP Rawalpindi for a bone marrow biopsy to confirm the diagnosis.

### 2.5. Bone Marrow Evaluation

**FIGURE 5:**
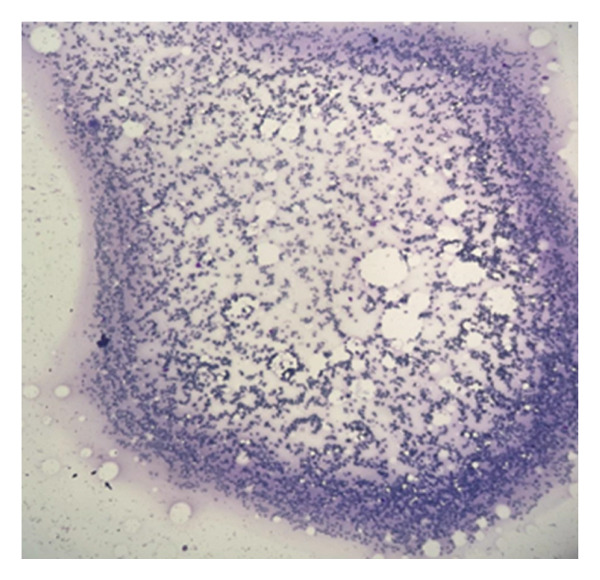
Bone marrow aspirate smear appearing diluted and hypocellular.

**FIGURE 7:**
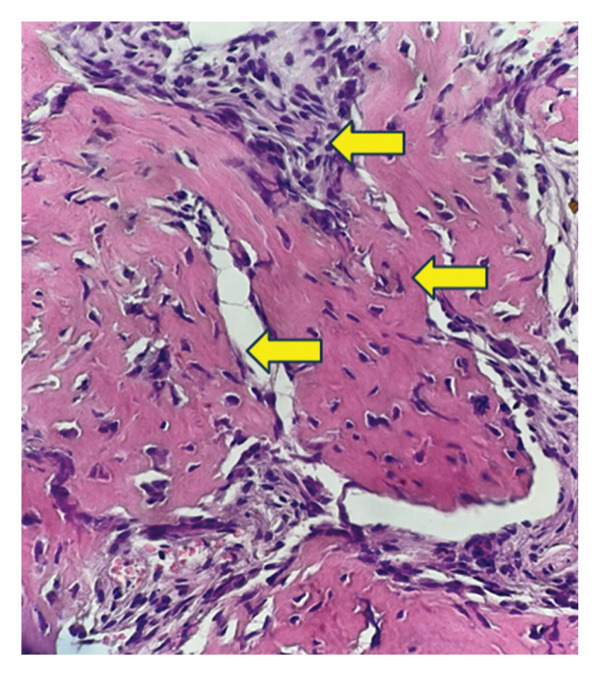
Higher‐magnification view showing fibrous stroma with poorly formed bony trabeculae lacking osteoblastic rimming.

**FIGURE 8:**
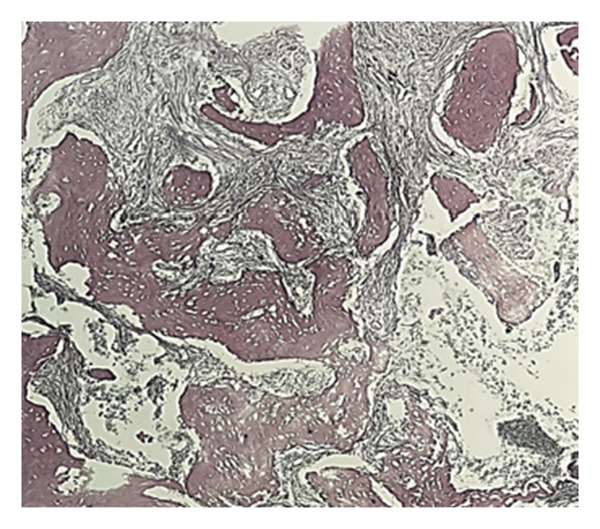
Reticulin staining showing Grade III myelofibrosis.

**FIGURE 9:**
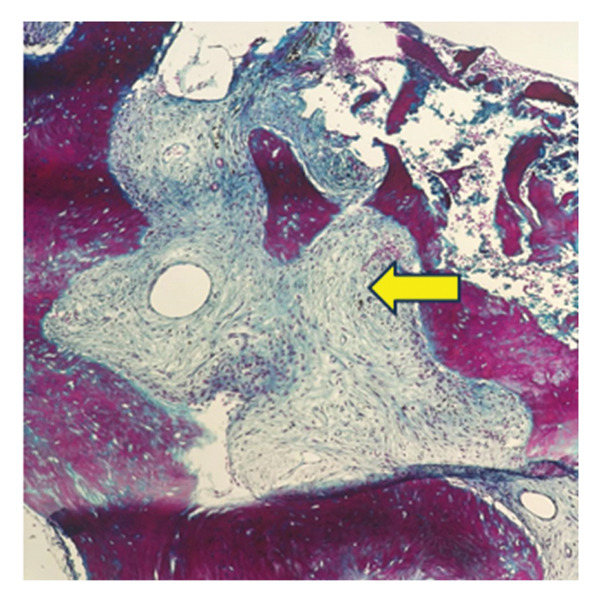
Masson trichrome stain highlighting dense collagen deposition (light blue) with fibrous stroma replacing marrow; residual skeletal muscle fibers stain pink.

Bone marrow aspirate showed diluted and hypocellular marrow (Figure 5). Trephine biopsy of the marrow showed extensive marrow fibrosis and near‐total replacement of hematopoietic tissue (Figure 6(a)). There were irregular trabeculae of woven bone, forming branching C‐ and S‐shaped patterns, characteristic of Chinese letter appearance, classic for FD (Figure 6(b)). At higher magnification, fibrous stroma with poorly formed bony trabeculae was seen, lacking osteoblastic rimming, another hallmark of FD (Figure 7). Grade III myelofibrosis was confirmed upon reticulin staining (Figure 8). Masson trichrome staining highlighted dense collagen deposition (blue) and fibrous stroma replacing the marrow, while residual skeletal muscle was stained pink (Figure 9). The key morphological differentiator between myelofibrosis of myeloproliferative neoplasms (MPNs) and myelofibrosis of FD is the presence of dysplastic megakaryocytes and myeloid hyperplasia in MPN, which is absent in FD, and the clonal nature of hemopoiesis in MPN, in contrast to reactive myelofibrosis in FD. In addition, the diagnosis of primary myelofibrosis in MPN is based on fulfilling the WHO diagnostic criteria based on morphology, cellularity, and genetic mutations (JAK2 and CAL‐R and MPL). However, in MS, there is co‐occurrence of FD along with IMs.

FIGURE 6Trephine biopsy sections showing (a) extensive marrow fibrosis with near‐total replacement of hematopoietic tissue and (b) irregular woven bone trabeculae with branching C‐ and S‐shaped configurations.

**Figure:**
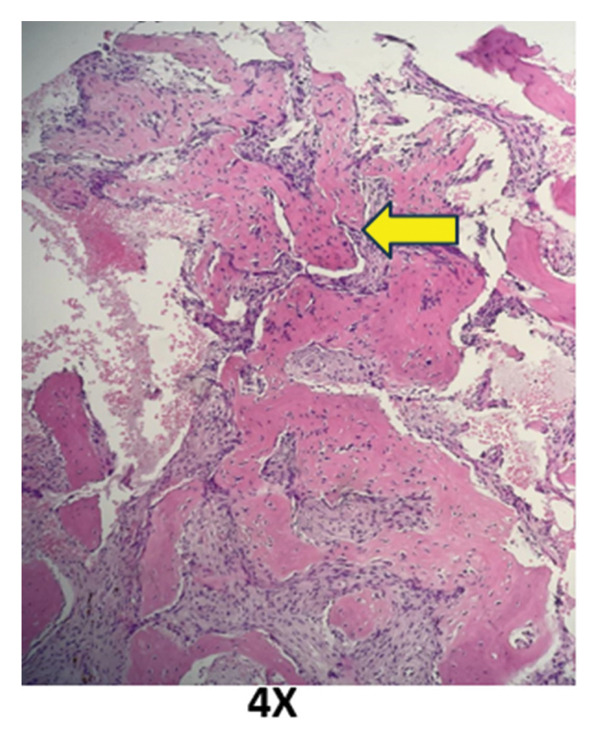
(a)

**Figure:**
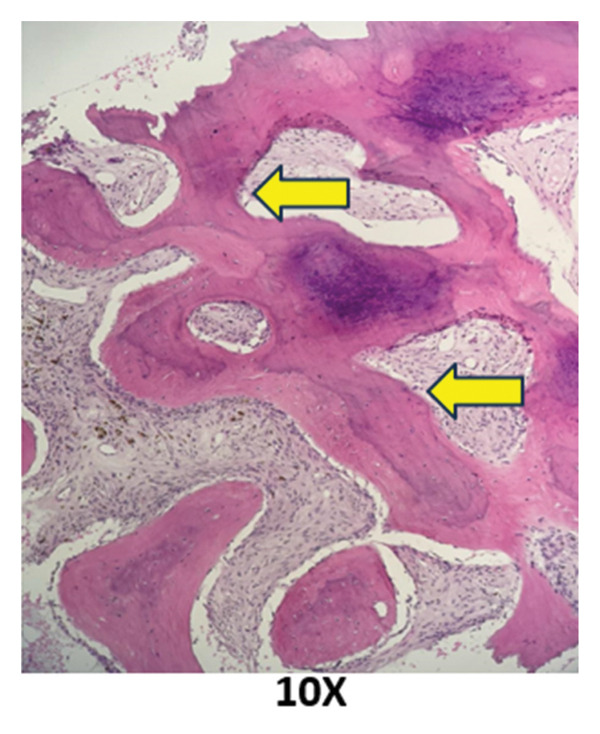
(b)

### 2.6. Biopsy

**FIGURE 10:**
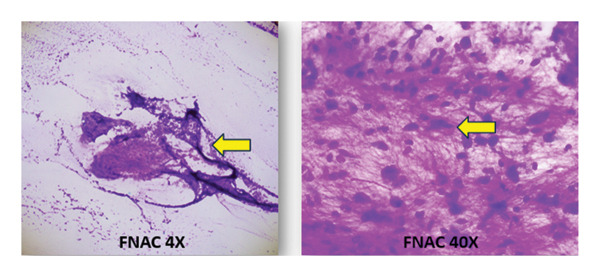
FNAC of the thigh lesion showing hypocellular smears with sparse spindle cells embedded in a myxoid matrix.

A biopsy was conducted, which confirmed the swelling to be a benign soft tissue myxoma. Fine‐needle aspiration cytology (FNAC) of the thigh lesion yielded hypocellular smears with sparse spindle cells embedded in a myxoid matrix, consistent with benign myxoma (Figure 10).

### 2.7. Diagnosis

Based on (i) the presence of multiple benign soft tissue myxomas, (ii) classic radiologic and histologic features of polyostotic FD, and (iii) marrow replacement with fibrotic and collagenous tissue, the patient was diagnosed with MS, a rare condition characterized by the coexistence of FD and IMs. Genetic testing for a GNAS mutation would have confirmed our diagnosis; however, due to the limited genetic testing facilities in Pakistan, the diagnosis was made based on clinical evaluation and the aforementioned investigations.

### 2.8. Treatment

Due to infection and severe deformity caused by myxomas in the thigh and groin, our patient underwent right leg amputation. He now uses a prosthetic limb, which has greatly improved his mobility. FD was managed conservatively with bisphosphonate therapy, without the need for surgical intervention. The patient also takes painkillers occasionally. He is currently on regular follow‐up every 6 months.

## 3. Discussion

MS is a rare concurrence of two relatively benign lesions: FD and IMs [9]. It has an estimated prevalence of < 1/1,000,000 [5] with approximately 107 cases reported globally to date [4]. To the best of our knowledge, this patient is the first documented case of MS in Pakistan, suggesting its unique nature and possible underdiagnosis of this condition due to insufficient resources or other diagnostic deficiencies, like genetic testing. It commonly affects females (68%) more than males (32%) [9]. Our case stands out with the diagnosis of a 64‐year‐old male, which varies from the observed global occurrence.

Despite being benign, FD can rarely undergo malignant transformation, most commonly into osteosarcoma, fibrosarcoma, chondrosarcoma, or malignant fibrous histiocytoma. This occurs in approximately 2.5% of the cases [10]. The incidence of malignant transformation is increased when FD occurs in association with MS, as reported in the literature [11]. However, there are only limited cases reporting any possible malignant transformation of IMs in MS patients, with only 4 cases reported by Zoccali et al. [7]. Crawford et al. recently reported a fifth case with the development of osteosarcoma in a fibrous dysplastic lesion in a patient with MS [12]. Despite occasional recurrences, IM is still considered a benign condition [4]. Because of frequent instances of recurrence reported in the literature, long‐term follow‐up is important [13].

The diagnosis of MS requires the identification of FD in combination with IM, regardless of whether the lesions are monostotic or polyostotic. Our study reports the presence of polyostotic lesions, which is comparable to other literature stating that the frequent occurrence of polyostotic lesions is more than monostotic lesions (3:1 ratio) [9]. In contrast to some previous reports stating no difficulty in walking and having no effect on daily activities [14], our patient struggled performing routine tasks due to extensive bony deformities, making him reliant on crutches. Reported cases have also shown associations with other conditions such as McCune–Albright syndrome, which may present with features like café‐au‐lait spots, early‐onset puberty, and thyroid abnormalities [4, 7, 15].

The exact etiology of the disease is not known, but previous literature revealed the pathogenesis of MS associated with GNAS1 activating R201 mutations [9]. Molecular genetic analysis has shown a missense mutation in the R201 position of the GNAS1 gene, which decreases GTPase activity and increases cAMP levels, and this was found after subjecting a patient to next‐generation sequencing (NGS) examination using the Variant Plex HS Solid Tumor kit [16]. Although the patient reported similar soft‐tissue swellings in first‐degree relatives (brother, son, and nephew), hereditary transmission of FD remains unproven. As GNAS mutations in FD are typically postzygotic somatic events rather than germ line, the familial clustering observed in our case may be coincidental or represent an unconfirmed genetic predisposition. Although genetic testing was not performed in this patient due to the limited availability of molecular diagnostics in our region, the final diagnosis was established based on concordant clinical presentation, radiologic findings, and histopathologic features. While GNAS mutation analysis could strengthen diagnostic certainty and may be valuable in future cases, especially for research and confirming suspected inheritance patterns, it was not essential for diagnosis in this case.

MS is typically managed with wide excision of myxomas when they cause discomfort or functional limitations [17]. In our patient, severe infection of the thigh and groin myxomas necessitated right leg amputation. The use of a prosthetic limb has since led to a marked improvement in mobility. Previous reports have noted local recurrence following incomplete excision, emphasizing the importance of regular follow‐up for both recurrence and new lesion development [18]. FD is usually treated with bisphosphonates, with surgery reserved for only select cases, as was done for our patient [19].

## 4. Conclusion

This case marks the first reported case of MS in Pakistan, highlighting the importance of clinical vigilance in patients with long‐standing bony deformities and soft tissue swellings. Despite the absence of genetic testing, a definitive diagnosis was made through a combination of imaging and histopathology. The patient’s severe disease burden and positive family history suggest a potential hereditary link although this cannot be confirmed in the absence of molecular testing. Early recognition and coordinated care are essential to prevent complications, reduce disability, and guide appropriate long‐term follow‐up.

## Author Contributions

Alizah Faisal: lead author and case conceptualization and manuscript writing. Asfand Yar Ali: clinical management, data acquisition, and manuscript writing. Hooria Waqas: radiological analysis, image curation, and manuscript writing. Hania Masood: literature review, background research, and manuscript writing. Muhammad Sheraz Hameed: manuscript writing and editing, Noor Ul Huda Al Hadi: pathophysiological commentary and clinical significance. Rahmat Gul Omarzai: reviewed and final approval of the manuscript.

Guarantor: Muhammad Sheraz Hameed.

## Funding

The authors received no financial support for the research, authorship, and/or publication of this article.

## Ethics Statement

No ethical approval was needed from the Ethical Committee.

## Consent

Informed consent was obtained from the patient to publish the material. The consent form is attached to a manuscript from the patient.

## Conflicts of Interest

The authors declare no conflicts of interest.

## Data Availability

The data used to support the findings of this study are available on request from the corresponding author.
